# Genomic insights into the domestication and genetic basis of yield in papaya

**DOI:** 10.1093/hr/uhaf045

**Published:** 2025-02-17

**Authors:** Min Yang, Xiangdong Kong, Chenping Zhou, Ruibin Kuang, Xiaming Wu, Chuanhe Liu, Han He, Ze Xu, Yuerong Wei

**Affiliations:** Institute of Fruit Tree Research, Guangdong Academy of Agricultural Sciences; Key Laboratory of South Subtropical Fruit Biology and Genetic Resource Utilization, Ministry of Agriculture and Rural Affairs, Guangdong Provincial Key Laboratory of Science and Technology Research on Fruit Tree, No. 80, Dafeng 2nd Street, Tianhe District, Guangzhou 510640, China; JiguangGene Biotechnology Co., Ltd., No. 9, Huida Road, Pukou District, Nanjing 210031, China; Institute of Fruit Tree Research, Guangdong Academy of Agricultural Sciences; Key Laboratory of South Subtropical Fruit Biology and Genetic Resource Utilization, Ministry of Agriculture and Rural Affairs, Guangdong Provincial Key Laboratory of Science and Technology Research on Fruit Tree, No. 80, Dafeng 2nd Street, Tianhe District, Guangzhou 510640, China; Institute of Fruit Tree Research, Guangdong Academy of Agricultural Sciences; Key Laboratory of South Subtropical Fruit Biology and Genetic Resource Utilization, Ministry of Agriculture and Rural Affairs, Guangdong Provincial Key Laboratory of Science and Technology Research on Fruit Tree, No. 80, Dafeng 2nd Street, Tianhe District, Guangzhou 510640, China; Institute of Fruit Tree Research, Guangdong Academy of Agricultural Sciences; Key Laboratory of South Subtropical Fruit Biology and Genetic Resource Utilization, Ministry of Agriculture and Rural Affairs, Guangdong Provincial Key Laboratory of Science and Technology Research on Fruit Tree, No. 80, Dafeng 2nd Street, Tianhe District, Guangzhou 510640, China; Institute of Fruit Tree Research, Guangdong Academy of Agricultural Sciences; Key Laboratory of South Subtropical Fruit Biology and Genetic Resource Utilization, Ministry of Agriculture and Rural Affairs, Guangdong Provincial Key Laboratory of Science and Technology Research on Fruit Tree, No. 80, Dafeng 2nd Street, Tianhe District, Guangzhou 510640, China; Institute of Fruit Tree Research, Guangdong Academy of Agricultural Sciences; Key Laboratory of South Subtropical Fruit Biology and Genetic Resource Utilization, Ministry of Agriculture and Rural Affairs, Guangdong Provincial Key Laboratory of Science and Technology Research on Fruit Tree, No. 80, Dafeng 2nd Street, Tianhe District, Guangzhou 510640, China; Institute of Fruit Tree Research, Guangdong Academy of Agricultural Sciences; Key Laboratory of South Subtropical Fruit Biology and Genetic Resource Utilization, Ministry of Agriculture and Rural Affairs, Guangdong Provincial Key Laboratory of Science and Technology Research on Fruit Tree, No. 80, Dafeng 2nd Street, Tianhe District, Guangzhou 510640, China; Institute of Fruit Tree Research, Guangdong Academy of Agricultural Sciences; Key Laboratory of South Subtropical Fruit Biology and Genetic Resource Utilization, Ministry of Agriculture and Rural Affairs, Guangdong Provincial Key Laboratory of Science and Technology Research on Fruit Tree, No. 80, Dafeng 2nd Street, Tianhe District, Guangzhou 510640, China

## Abstract

Papaya (*Carica papaya* L.) is an important tropical and subtropical fruit crop, and understanding its genome is essential for breeding. In this study, we assembled a high-quality genome of 344.17 Mb for the newly cultivated papaya ‘Zihui’, which contains 22 250 protein-coding genes. By integrating 201 resequenced papaya genomes, we identified four distinct papaya groups and a 34 Mb genomic region with strong domestication selection signals. Within these regions, two key genes associated with papaya yield were discovered: *Cp_zihui06549*, encoding a leucine-rich receptor-like protein kinase, and *Cp_zihui06768*, encoding the accumulation of photosystem one 1 (APO1) protein. Heterologous expression of *Cp_zihui06549* in tomato confirmed that the total number of fruits in transgenic lines more than doubled compared to wild-type plants, resulting in a significant yield increase. Furthermore, we constructed a pan-genome of papaya and obtained a 77.41 Mb nonreference sequence containing 1543 genes. Within this pan-genome, 2483 variable genes, we detected, including four genes annotated as the ‘terpene synthase activity’ Gene Ontology term, which were lost in cultivars during domestication. Finally, gene retention analyses were performed using gene presence and absence variation data and differentially expressed genes across various tissues and organs. This study provides valuable insights into the genes and loci associated with phenotypes and domestication processes, laying a solid foundation for future papaya breeding efforts.

## Introduction

Papaya (*Carica papaya* L.) is an important economic fruit crop belonging to the family *Caricaceae*. It originated in the southern Mexico and Costa Rica [1], and is now distributed in tropical and subtropical regions around the world. It is a fast-growing, dicotyledonous, polygamous species with a small genome of nine pairs of chromosomes (2*n* = 18) [1, 2]. Papaya has a high nutritional and medicinal value [3]. For example, papaya fruit is rich in vitamins A and C, folate, iron, and calcium [4] and is an excellent source of beta-carotene, which may help prevent cancer, diabetes, and heart disease [5]. Unripe green fruits can be used as vegetables and is also a source of papain, an endocytic plant cysteine protease used as a meat tenderizer for thousands of years [6, 7]. Papaya is also used in the pharmaceutical and cosmetics industries [1]. Global papaya production has increased steadily over the past twenty years by about 4.35% annually [8].

**Figure 1:**
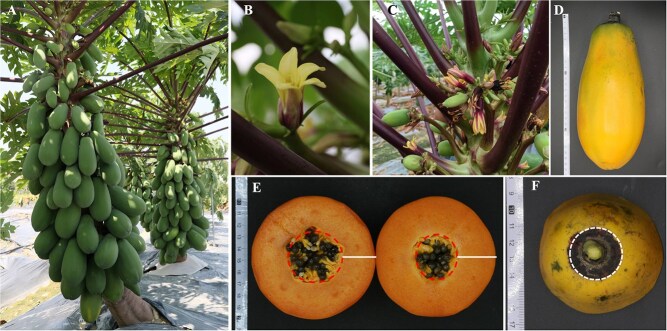
The papaya cultivar Zihui with high quality and high yield. A. The whole plant morphology of ‘Zihui’ papaya. B. ‘Zihui’ has yellow flowers. C. inflorescence and flower quantity. D. Mature papaya fruit. E. The inner cavity and flesh thickness of papaya fruit, (left) common papaya cultivar, (right) ‘Zihui’ papaya. F. The fruit shoulder of ‘Zihui’ papaya

Like other domesticated species, papaya has a significantly reduced genetic variation compared to its wild ancestors due to population bottlenecks and strong artificial selection during domestication [9]. Over the past century, the shift from traditional accessions to uniform elite strains has further weakened papaya’s genetic diversity and constrained breeding efforts. As a result, researchers are increasingly interested in exploring the genetic variation present in papaya germplasm resources [10, 11]. Papaya germplasm exhibits considerable phenotypic variation for many horticulturally important traits, including: size, shape, flesh color, flavor, sweetness, juvenile period duration, plant stature, stamen carpellody, and carpel abortion [10]. Until recently; however, research on papaya germplasm resources has been limited, with little focus on the genetic differences of various local resources or ecotypes and the genetic diversity of existing accessions. Understanding of the papaya germplasm is mainly focused on external morphology and horticultural characteristics, with few in-depth studies performed at the cytogenetic and molecular genetic levels. This has led to a blindness in selecting hybrid parents, inefficiencies in breeding, and an ineffective use of germplasm resources. In addition, the lack of uniform naming methods for papaya accessions in different regions has confused and hindered communication and collaboration in breeding efforts. Subjective deviations in the classification of papaya accessions due to the absence of molecular data standards have also been an issue. Germplasm sequencing of crop plants has provided valuable insights into global genetic variation distribution and has helped accelerate the breeding process for some crops [12, 13]. Although genome assembly of two accessions and resequencing of some germplasm resources on papaya have been completed, and preliminary analysis of sex differentiation, disease resistance, and differences between transgenic and nontransgenic materials has been conducted based on these data [14, 15]. Currently, however, there is little research on the genomes of the main, local, cultivated papayas, and the genomes of these accessions are often highly heterozygous and difficult to assemble. Nevertheless, analyzing the genomes of cultivated papaya is important for mining and using beneficial genes and conducting rapid molecular breeding.

‘Zihui’ papaya is a major cultivar in southern China, which is popular for its high quality, high yield, thick flesh, strong resistance, and high vitamin C content [16]. To explore the valuable genes of this cultivar, obtaining its chromosomal-level genome is essential. Therefore, this study presents the genome assembly of ‘Zihui’ through PacBio HiFi and Hi-C sequencing technologies. Among its 22 250 genes, 283 are specific to ‘Zihui’, compared to cultivars ‘Sunset’ and ‘Sunup’. In addition to obtaining resequencing data on papaya from previous studies [14, 15, 17], we resequenced 49 papaya samples and identified 2.36 million high-quality single-nucleotide polymorphisms (SNPs). We scanned the selection signals during papaya domestication, conducted genome-wide association studies (GWAS) on fruit weight and yield, and identified some loci associated with fruit growth and stress resistance. Further, we constructed the papaya pan-genome, conducted selection analyses based on gene presence and absence variation (PAV), and integrated them with papaya expression profiles for gene retention analysis. Our results provide new insights and a foundation for papaya domestication and breeding research.

## Results

### ‘Zihui’ papaya

‘Zihui’ (Plant variety number of the Ministry of Agriculture and Rural Affairs of the People’s Republic of China: CNA20201005052; Evaluation number of nonmajor crop varieties in Guangdong province, China: 20210009), a new and major cultivated papaya of high quality and high yield in southern China, which was selected from the hybrid progeny of Taiguohong as the female parent and the small-fruit Hawaiian type GZ201301301 as the male parent by the Guangdong Academy of Agricultural Sciences in China [16]. ‘Zihui’ papaya exhibits stable genetic features and is characterized by strong adaptability, vigorous growth, a low-fruiting position, excellent commercial fruit traits, high yield, and superior quality. The stems of this cultivar begin to bear fruit at a height of approximately 34.5 centimeters above the ground, with a large number of flowers and a strong continuous fruit-setting rate. The effective yield per plant is about 64.4 kg, with a high yield of about 116 tons per hectare. The fruit is long, oval in shape, with a purple ring on the shoulder. The flesh is orange-red in color, thick, and juicy. The weight of a single fruit is about 1142.7 g, with a soluble solids content is 11.5 g 100 g^−1^, the vitamin C content is 1.01 mg·g^−1^, and the crude fiber content is 0.6 g·100 g^−1^ (Fig. 1, Table 1).

**Table 1:** Characteristics of yield and fruit quality of ‘Zihui’ papaya.

Stems start to bear fruit (cm)	Average single fruit Weight (g)	Yield of single plant (Kg)	Yield per hectare (tons)	Soluble solids content (g·100 g^−1^)	Vitamin C content (mg·g^−1^)	Crude fiber content (g·100 g^−1^)
34.53 ± 7.54	1142.70 ± 23.23	64.40 ± 6.44	115.92 ± 11.59	11.50 ± 0.5	1.01 ± 0.01	0.60 ± 0.05

### Genome and evolutionary analysis of ‘Zihui’ papaya

**Figure 2:**
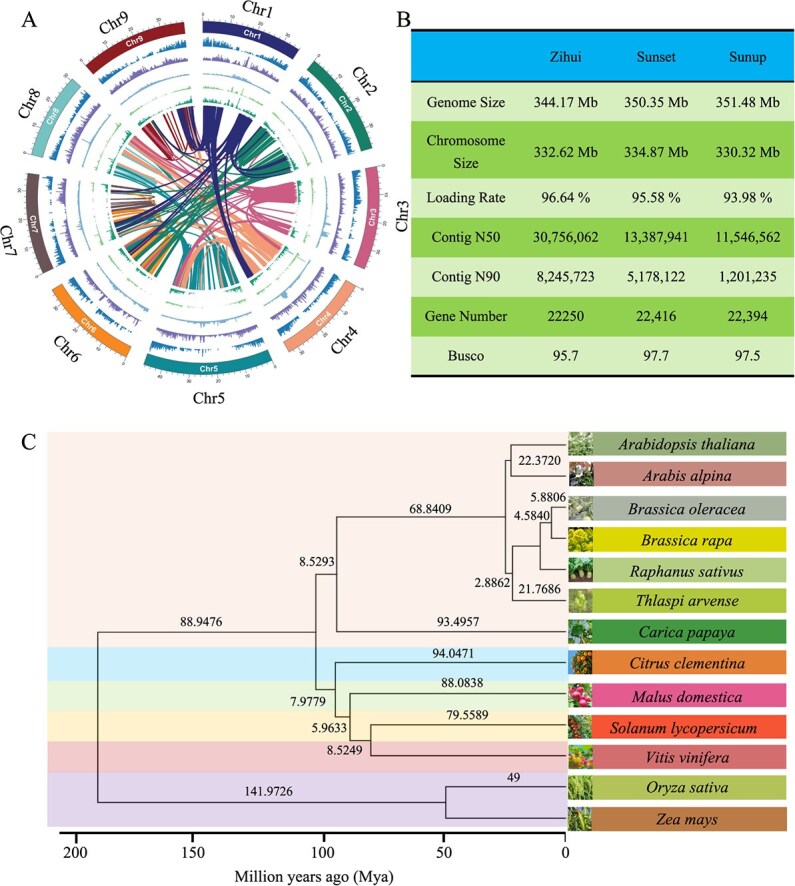
The genome of ‘Zihui’ papaya. A. Circos plot showing the genome features, with the outermost circle representing chromosomes, followed in turn by the density of protein-coding genes, SNP distribution, transposon density, synonymous mutation density, and nonsynonymous mutation density. Internal connections represent gene collinearity. B. Overview of genome assembly. C. Multispecies phylogenetic tree and divergence time based on single-copy genes

To gain deeper insights into the genetic basis of papaya traits and provide a high-quality resource for future research, we conducted a comprehensive genome analysis of hermaphroditic plants of the ‘Zihui’ papaya. This study generated 10.29 Gb (~30 ×) of PacBio HiFi long reads data that were used to perform a preliminary genome assembly of the papaya. This resulted in a 344.26 Mb draft genome with an N50 of 30.76 Mb. After two rounds polishing the PacBio HiFi long reads data and three rounds of NGS data polishing, an improved genome of 344.17 Mb was obtained with a heterozygosity rate of 0.5%. Using the genomes of ‘Sunup’ and ‘Sunset’ as references, the contigs were oriented and conflicts were resolved using Hi-C sequencing data (~100×), resulting in a final genome of 344.17 Mb, comprising nine pseudomolecules and 177 contigs. Benchmarking Universal Single-Copy Orthologs (BUSCO) analysis of the genome indicated that 95.7% of core eukaryotic genes could be identified. Repeat annotation showed that 206 Mb (59.89%) of the genome consisted of repetitive sequences (Fig. 2A). Based on *ab initio* gene prediction, homology-based gene prediction, and transcriptome data for gene model prediction, 22 250 coding protein genes were predicted in the genome (Fig. 2B). Compared to the ‘Sunup’ and ‘Sunset’ genomes, 283 genes specific to the ‘Zihui’ genome represent unique genetic resources of ‘Zihui’ (Supplementary Fig. S1B). These Zihui-specific genes were mainly enriched in GO terms such as ‘embryo sac central cell differentiation’ and ‘MAPK cascade’ (Supplementary Fig. S1D). With more papaya phenotypes being studied, these genes will become valuable resources.

Papaya, along with model species such as *Arabidopsis thaliana* and *Brassica rapa*, belongs to the order Brassicales. Previous studies have also revealed the phylogenetic relationship between papaya and other species within this order [18, 19]. However, as genomic data accumulate, more genomes in the Brassicales have become accessible, so that further analysis can be conducted using the genomes of new species to enhance our understanding of the evolutionary relationships among the order. Based on the protein sequences of *Brassica rapa*, *Citrus clementina*, *Malus domestica*, *Solanum lycopersicum*, *Vitis vinifera*, *Oryza sativa*, and *Zea mays*, 72 845 ortholog groups were identified, among which 15 496 contained papaya genes. A phylogenetic tree was constructed based on 219 single-copy genes from 13 species. Estimation of the divergence time of these species using BREAST2 revealed that the divergence time between papaya and other *Brassicales* was relatively ancient (Fig. 2C), with the divergence between papaya and *Citrus clementina* occurring about 102.025 Mya. Although papaya belongs to the *Brassicales*, its morphology and characteristics are very different from those of other species in the family, such as Arabidopsis and *Brassica oleracea*, possibly due to their early divergence.

### Population structure analysis

Understanding the genetic diversity and population structure of papaya is crucial for uncovering the genetic basis of its domestication and breeding. Wild papaya, as a reservoir of untapped genetic diversity, holds significant potential for improving cultivated varieties. Therefore, we analyzed the phylogenetic relationships and population structure of wild and cultivated papaya accessions to identify key genetic resources and evolutionary patterns. In this study, we sequenced 49 papaya accessions, with sequencing depths ranging from 8.2 to 10.5 × (Supplementary Table S1). We also obtained whole genome sequencing data of 152 samples from the National Center for Biotechnology Information (NCBI), including 49 wild and 103 cultivated papaya accessions (Supplementary Table S2). A total of 2 363 973 SNPs were identified in the papaya population, of which 76 621 were in the coding regions. Functional annotation classified these SNPs into 16 types, including upstream gene variants, synonymous variants, stop retained variants, stop lost, and stop gained. Among them, 39 095 were missense mutations, and 36 460 were nonsense mutations. These SNP data provide a valuable resource for papaya breeding research.

**Figure 3:**
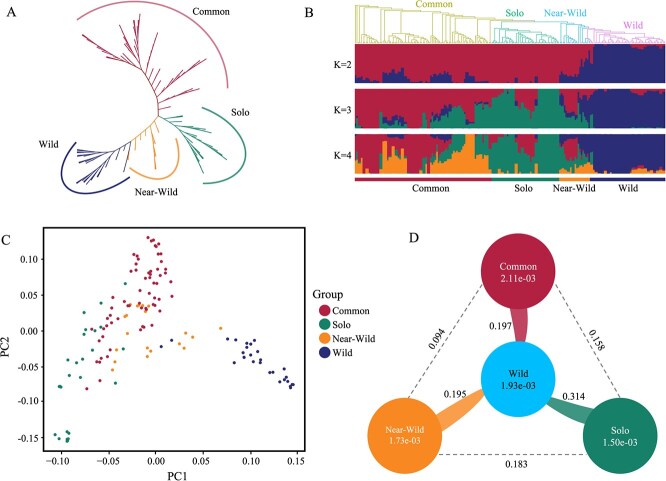
Population structure analysis of papaya. A. Phylogenetic tree of papaya populations. B. Population structure plot of papaya individuals. C. Principal component analysis plots of papaya accessions based on SNPs. D. Fst values between the common, solo, near-wild, and wild papaya populations, as well as π values for each of these four populations

Using these high-quality SNPs, we constructed the phylogenetic relationships of papaya, revealing that the papaya population could be mainly divided into four groups (Fig. 3A). The common group contained 89 individuals with common accessions; the solo group contained 44 individuals with solo accessions; the wild group contained all wild accessions; and the ‘near-wild’ group contained 20 individuals with unique, special local accessions that could be clustered with neither the solo group nor the wild group. The population structure analysis based on SNPs (Fig. 3B) showed that the wild population had not been affected by hybridization or gene flow, while the common, solo, and near-wild groups had undergone extensive hybridization, consistent with papaya breeding practice. PCA analysis based on SNPs (Fig. 3C) showed that the wild population and the cultivated population were significantly different in the first two principal components, while some accessions among the cultivated accessions had similar relationships due to hybridization.

We evaluated the nucleotide diversity (π) of the wild, common, solo, and near-wild populations separately (Fig. 3D). The common population had the highest π of 2.11e^−3^, while the solo population had the lowest π of 1.5e^−3^. Compared with the π value (1.93e^−3^) of the wild papaya population, the common population did not have a domestication bottleneck, while the solo did, indicating that different domestication processes might have different effects on the domestication bottleneck of papaya.

Calculation of the fixation index (Fst) showed that the genetic differentiation between the solo population and the wild papaya was the greatest (0.314). In contrast, genetic differentiation between the common and near-wild populations and between the common and wild papaya was relatively close, with Fst values at 0.094 and 0.197, respectively. These results further indicated that the solo population might have experienced a greater domestication bottleneck than the common population, providing important resources for further evaluating the genetic relationships among papaya germplasms and understanding genetic differentiation during domestication.

### GWAS deciphered a leucine-rich receptor-like protein kinase that conferred yield increase

**Figure 4:**
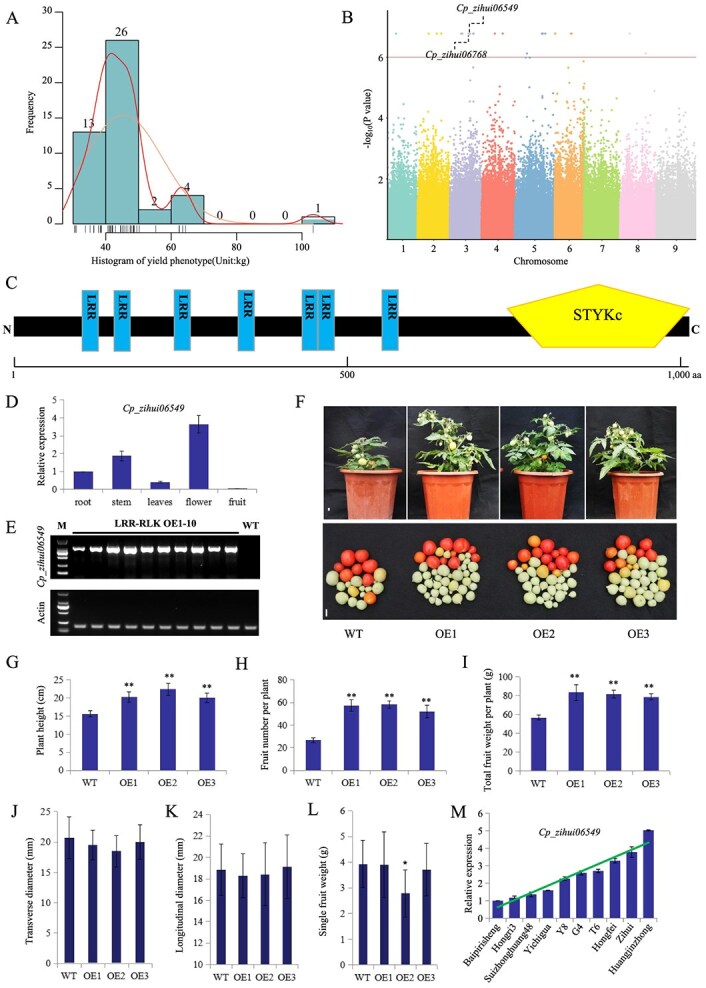
GWAS analysis and biological function verification of candidate genes. A. Frequency and density distribution of papaya plants with different yields. B. Manhattan plot of GWAS for papaya yield. C. Schematic diagram of the Cp_zihui06549 protein structure. D. Expression patterns of *Cp_zihui06549* in papaya tissues. E. Expression of *Cp_zihui06549* in leaves of WT and 10 independently transformed homozygous transgenic plants were detected by RT-PCR. F. Comparison of phenotypes of the WT plant and the Cp_zihui06549-OE transgenic plants. Scale bar, 1 cm. G–L, plant height (G), fruit number per plant (H), total fruit weight per plant (I), transverse diameter (J), longitudinal diameter (K) and single fruit weight (L) in WT and transgenic plants. WT, the wild-type cultivated tomato ‘Micro Tom’; OE, the overexpression transgenic lines of *Cp_zihui06549.* M. Expression level of *Cp_zihui06549* in leaves of 10 papaya varieties with different yields. Mean values ± SDs are shown for three biological replicates (*n* = 3); ^*^P < 0.05, ^**^P < 0.01

Domestication and breeding efforts have resulted in significant phenotypic variations in papaya, particularly in traits like fruit weight and yield (Supplementary Table S3). Papaya fruit yield ranged from 30.4 kg in accession BG to 103.4 kg in the accession ‘Huangjinzhong’ (Fig. 4A). By identifying genetic factors underlying these variations, we can better understand the evolutionary pressures and mechanisms driving papaya improvement. This study measured the papaya yield (total fruit weight per tree and single fruit weight) in 2019 and 2020. Using 2 363 973 SNPs spanning the entire papaya genome, a GWAS was performed on papaya yield using the mixed linear model (MLM) approach. GWAS with a suggestive *P*-value threshold of 10^−6^ identified 55 SNPs associated with total fruit weight per tree (Fig. 4B, Supplementary Fig. S2). These significant SNPs were located on chromosomes 1, 2, 3, 4, 5, 6, and 8. Among these loci, only five are situated within the promoter regions, while the others are located in the intergenic region. And these five loci are located in the promoter of the *Cp_zihui06549* (leucine-rich receptor-like protein kinase family protein) and *Cp_zihui06768* (accumulation of photosystem I, APO1) (Supplementary Table S4). These findings suggest that these two genes may have played a key role in yield improvement during papaya domestication. In addition, GWAS also identified four SNPs (chr6: 20993174, chr6: 20993180, chr9: 26800072, chr9: 26800081) located in intergenic regions that were significantly associated with the single fruit weight. Although these significant SNPs cannot be directly linked to specific genes, these results suggest potential regulatory elements or distal genetic regions that may influence fruit weight, providing new insights into the genetic architecture of this important trait.

To further confirm the GWAS results, we selected *Cp_zihui06549* for functional verification. *Cp_zihui06549* encodes a protein belonging to the leucine-rich receptor-like protein kinase (LRR-RLK) family (Fig. 4C). *Cp_zihui06549* was preferentially expressed in flowers and stems (Fig. 4D). We also expressed *Cp_zihui06549* in the wide-type (WT) tomato cultivar ‘Micro-Tom’ under control of the CaMV 35S promoter. In total, 10 independently transformed homozygous transgenic plants were obtained that expressed *Cp_zihui06549* in varying degrees (Fig. 4E). Subsequently, three lines of LRR-RLK OE1–3 were selected for further experiments. Compared to WT plants, transgenic lines were taller, with more lateral branches, the total number of fruits increased by more than 2-fold, with no significant changes in the transverse or longitudinal diameters of the fruits, resulting in a significant increase in yield (Fig. 4F-K), only OE2 had slight reductions in single-fruit weight (Fig. 4L). At the same time, 10 varieties with different yields were randomly selected and the expression level of *Cp_zihui06549* in leaves was detected by quantitative real-time polymerase chain reaction (qRT-PCR), results showed that the expression of *Cp_zihui06549* was higher in high-yielding papaya varieties than in low-yielding varieties (Fig. 4M). This was consistent with the phenotype results of these varieties (Supplementary Table S3). Therefore, this gene represents a promising target for increasing yield or biomass through papaya breeding.

### Papaya pan-genome construction

**Figure 5:**
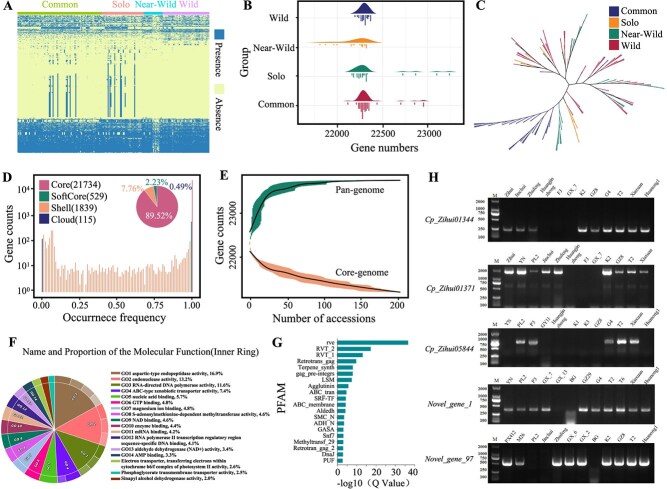
Pan-genome construction of *C. papaya*. A. Heatmap of gene PAV, where yellow represents absence, and blue represents presence. B. Number of genes in four *C. papaya* populations. Some samples in the near-wild population have fewer genes, while some samples in the solo and common populations have more genes. C. Phylogenetic tree constructed based on binary gene PAV data. D. Distribution of the number of core genes, softcore genes, shell genes, and cloud genes. E. Fitting graph of the total gene number and core gene number in the pan-genome, with 100 random samplings for each sample-size node. F. GO enrichment results of shell genes. G. Pfam enrichment analysis of shell genes. H. PCR amplification of five shell genes in different accessions

The use of a single reference genome does not capture diversity present among different papaya cultivars. Constructing a pan-genome provides a comprehensive view of genetic variation, capturing novel sequences and genes that are absent in the reference genome. Therefore, our study constructed the first papaya pan-genome based on the 201 papaya resequencing genomes. By assembling unmapped reads, 590 Mb of sequences were obtained. After filtering out redundancy and contamination, we obtained 77.41 Mb of nonreference sequences. Through genomic structure prediction, 1530 genes were identified in the nonreference sequence. Since hybridization between different papaya genomes could lead to gene exchange, hybrid accessions may have more genes than their parents. Based on the gene PAVs in the pan-genome, cultivated papaya-specified genes were identified (Fig. 5A). The distribution of gene numbers in different papaya populations showed that the common and solo groups, which were often hybridized during breeding, had the most genes (Fig. 5B). Construction of a phylogenetic tree based on gene PAV (1 indicating presence and 0 indicating absence) revealed a different relationship compared to the tree constructed using SNPs (Fig. 5C).

According to a previously described classification method [20], genes present in 100% of individuals were defined as core genes, those present in 99%–100% of individuals as softcore genes, those present in 1%–99% of individuals as shell genes, and those present in less than 1% of individuals as cloud genes. Using this classification standard, the papaya population contained 21 734 core genes, 529 softcore genes, 1839 shell genes, and 115 cloud genes (Fig. 5D). The number of core genes decreased in the papaya population as the number of samples increased (Fig. 5E). The total number of genes in the pan-genome approached saturation in the papaya group with more than 100 individuals. Variable genes in the population were lost or gained during domestication, and their functions may be related to the traits selected by artificial selection. GO enrichment analysis showed that shell genes were enriched in many GO terms (Fig. 5F, Supplementary Fig. S3, Supplementary Table S5), some of which were related to fruit ripening or to metabolites, such as ‘S-adenosylmethionine-dependent methyltransferase activity’ [21]. There were 62 genes in the pan-genome belonging to this GO term, of which 23 were variable genes, and 38 were located in the reference genome. Based on the NCBI NR database annotation, these genes mainly encode proteins like S-adenosyl-L-methionine-dependent methyltransferase and 2-methoxy-6-polyprenyl-1,4-benzoquinol methylase, which are associated with metabolic features in fruits [22]. Another significantly enriched GO term, ‘MAPK cascade’ is a chain reaction of protein kinases activated in response to environmental stress, biological processes, growth, and development. There were 68 genes in this GO term, of which nine were located in the nonreference sequence. The pfam enrichment analysis also revealed that shell genes were enriched in some genes associated with biotic stress (Fig. 5G), such as terpene_synth (terpene synthase N-terminal domain-containing protein). This study also validated the accuracy of gene PAV through PCR, which is crucial for downstream analysis. We randomly selected five shell genes and performed PCR validation on 12 randomly chosen varieties, which corresponded with the results of gene PAV calling (Fig. 5H).

### Selection signal analysis of papaya

**Figure 6:**
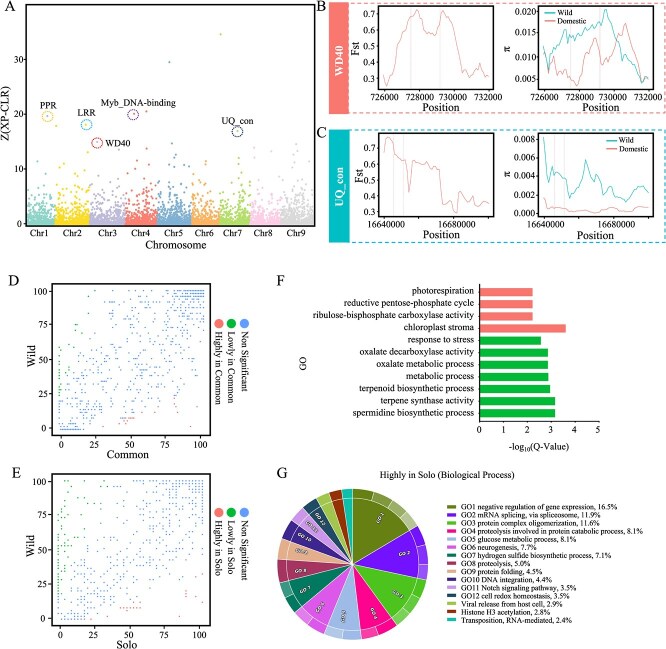
Selection signal analysis of papaya populations. A. Manhattan plot of *z*-score values obtained from XP-CLR analysis of selection signals between the cultivated and wild papaya populations. Circled points indicate genes with domains (annotated from the pfam database) that have selection signals in the top 5% of the population’s genome. Genes labeled on the plot have their domains annotated. B and C. Fst and π values of the *Cp_zihui05794* gene encoding a protein with the W40 domain (B) and the *Cp_zihui15185* gene encoding a protein with the UQ_con domain (C) between the wild and cultivated populations. The vertical dashed lines on the *x*-axis indicate gene boundaries, and the region between the dashed lines represents the gene region. The dashed lines on either side represent the upstream and downstream regions of genes, respectively, extending 4 kb from the boundaries. D. Comparison of gene frequency between the common and wild populations based on gene PAV, indicating high-frequency and low-frequency genes in the common populations (fold difference in frequency > 2, p.adj <0.001). E. Comparison of gene frequency between the solo and wild populations based on gene PAV, indicating high-frequency and low-frequency genes in the solo population. F. GO enrichment entries of low-frequency or high-frequency genes in common populations (hypergeometric distribution test, p.adj <0.05). G. GO enrichment analysis of genes with frequency highly in Solo

During the domestication process, important traits in plants undergo changes. For example, the fruit size of cultivated papayas is larger than that of the wild type due to domestication and improvement, resulting in an increase in fruit weight. To identify domestication selection signals associated with these important agricultural traits, we scanned genomic regions and identified regions with XP-CLR scores in the top 5% (Fig. 6A). In this study, we identified 392 selection signal regions in the genome, ranging in length from 10 to 480 kb, covering a total of 34 Mb of the chromosome regions, and including 1185 genes (Supplementary Table S6). Many of these regions contained genes with potentially important biological functions. For instance, the *Cp_zihui05794* gene on chromosome 3, which encodes a protein with a WD40 (pfam number: PF00400) functional domain, plays an important role in multiple biological pathways [23, 24]. The *Fst* value calculation revealed that this gene region had a significant genetic difference between the domesticated and wild populations (Fig. 6B), further confirming the results of XP-CLR analysis. Additionally, in the wild population, the gene region and its upstream 4 kb region (promoter region) had a higher π value than the cultivated population. The selection signal region on chromosome seven contained the *Cp_zihui15185* gene, which encodes the ubiquitin-conjugating enzyme (UQ_con), an important gene in the papaya fruit ripening process [25]. The π value of this gene was very low in the cultivated population (Fig. 6C) and significantly different between the cultivated and wild populations, indicating a reduction in diversity after domestication. Other important genes in the selection signal regions include the *Cp_zihui01752* gene encoding a pentatricopeptide repeat (PPR) protein and the *Cp_zihui04511* gene encoding a leucine-rich repeat (LRR) domain-containing protein, both of these genes are essential for the biological resistance of papaya [26, 27].

Although gene frequency differences between populations are not solely due to selection but can also be due to genetic drift or hybridization, analyzing the differences in gene frequency between cultivated and wild populations can help understand the impact of selection on gene PAVs. In this study, the gene frequency of common and solo populations was compared to the wild population. The numbers of genes with low frequency were 29 and 367 for the common and solo populations, respectively, while those with high frequency were 36 and 110, respectively, for the common and solo populations (Fig. 6D and E, Supplementary Table S7). These results indicated that more genes were lost than gained during domestication, emphasizing the importance of identifying lost genes from the wild population. GO enrichment analysis of genes with significant frequency differences revealed that genes related to resistance, such as ‘response to stress’ and ‘terpene synthase activity’, were enriched in the low-frequency genes of the domesticated population (Fig. 6F, Supplementary Fig. S4). In papaya’s pan-genome, 10 genes related to ‘terpene synthase activity’ were identified, of which two were lost in common and two in solo populations. This implied that genes related to resistance may have been lost in the domestication process due to focusing only on enhancing productive traits such as yield. Although the number of high-frequency genes in the solo population was smaller than that of low-frequency genes, they were significantly enriched in many GO terms (Fig. 6G, Supplementary Fig. S4). Endogenous hydrogen sulfide in plants plays an important role in regulating fruit ripening and quality changes [28]. Genes with high frequency in the solo population were enriched in the ‘hydrogen sulfide biosynthetic process’ GO term (Fig. 6G), indicating that the ripening process and quality in papaya fruits were important considerations during domestication. Additionally, three gene PAVs in this GO term were under selection, suggesting these genes may play an essential role in improving the quality and productivity of papaya fruits.

### Gene expression atlas and gene retention analysis

**Figure 7:**
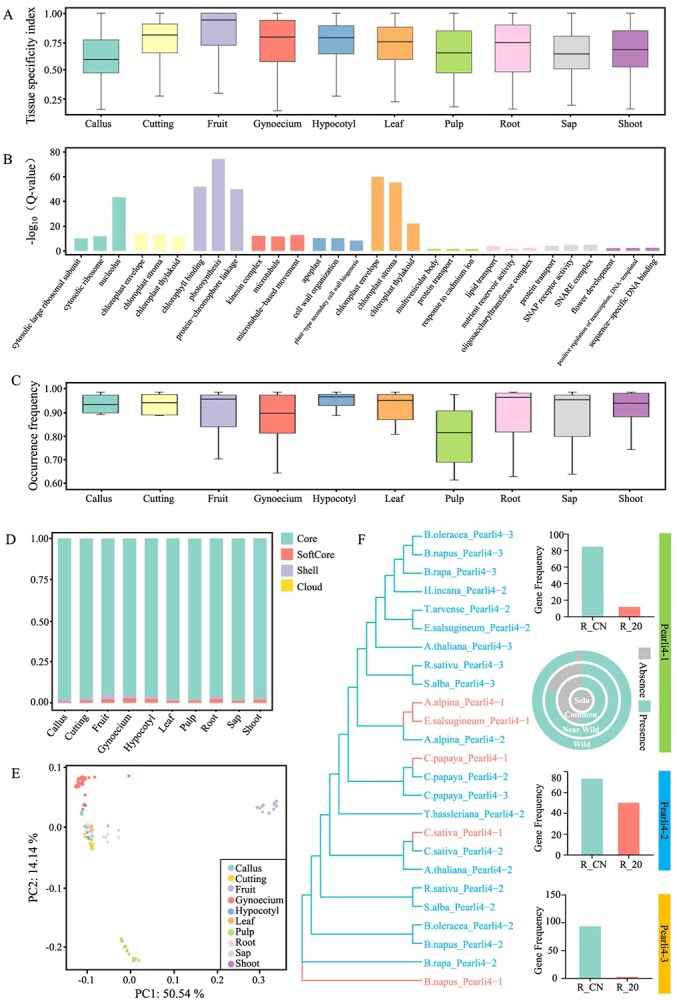
Gene expression and gene retention analysis in papaya. A. Tissue specificity index value distribution of genes with organ or tissue-specific expression. B. GO terms enriched in genes with organ or tissue-specific expression. C. Gene frequency distribution of genes with organ or tissue-specific expression in papaya populations. D. Proportion of core, softcore, shell, and cloud genes among genes with organ or tissue-specific expression. E. PCA analysis of gene expression in multiple tissues or organs. F. Phylogenetic tree constructed based on homologous genes of phospholipase-like protein4 1-3 in papaya and other species, and the expression levels of genes encoding these proteins in different samples. Pearli4-1 is a shell gene, and the circular graph shows the frequency of genes encoding this protein in different papaya populations

Exploring gene expression patterns can provide insights into the functional diversity of papaya genes and their domestication history. Analyses of RNA-seq data from multiple tissues and organs of papaya conducted in this study revealed the tissue-specific expression patterns of genes in papaya. PCA analysis of gene expression showed that the expression levels of genes in gynoecium, fruit, and pulp were less similar to those in other parts (Fig. 7E). Among all the tested organs and tissues, fruit had the highest gene expression specificity index, with 1223 specifically expressed genes (Fig. 7A), while callus tissue had the lowest gene expression specificity index, possibly because callus is composed of undifferentiated cells and lacks strong tissue-specific expression patterns. Tissue-specific expressed genes (Supplementary Table S8) in fruit and leaf were enriched in photosynthesis-related GO terms, such as ‘protein-chromophore linkage’ and ‘chlorophyll binding’ in fruit, and the ‘chloroplast envelope’ in leaves (Fig. 7B). Furthermore, by integrating gene PAV information, we showed that the conservation of tissue-specific expressed genes differed among different organs. The proportion of shell genes among the tissue-specific expressed genes was highest in fruit (Fig. 7D), further indicating that genes related to various fruit traits may have undergone more selection during the domestication process, resulting in more gene PAVs. Analysis of the distribution of tissue-specific expressed genes in the papaya population showed that the pulp had the lowest frequency of tissue-specific expressed genes (Fig. 7C), which may be related to fewer tissue-specific expressed genes in the pulp.

Plant gene and genome evolution is primarily driven by gene duplication, which can be observed in all plant genomes. However, not all paralogous genes generated by gene duplication persist in plant genomes for extended periods, and many are lost due to genetic drift. We identified a class of genes encoding phospholipase-like proteins that all contained the PEARLI-4 protein functional domain (pfam number PF05278). Three of these genes were found to be adjacent on chromosome 4 from 25.64 to 25.67 Mb, suggesting they arose from tandem duplication (Fig. 7F). Sequence alignment with 11 other species, including *Brassica oleracea, B. napus, B. rapa, Hirschfeldia incana, Thlaspi arvense, Eutrema salsugineum, Arabidopsis thaliana, Raphanus sativus, Salix alba, Arabis alpine,* and *Camelina sativa*, revealed that the Pearli4–1–3 gene was present in 11 species (Fig. 7F), with only four species having the Pearli4–1 gene. This suggested that Pearli4–1 might have duplicated after the divergence of papaya from most of the Brassicales, making it the most recently duplicated gene among Pearli4–1, Pearli4–2, and Pearli4–3. Interestingly, all three genes in papaya were responsive to drought stress for 20 days, indicating functional redundancy. Pearli4–1 might be the most recently duplicated gene and, as a shell gene, more likely to be lost, consistent with the finding by Jia *et al.* (2023) in barley. We also found that the absence rate of this gene was very low in wild papaya but very high in the cultivated common and solo populations, suggesting that artificial selection pressure likely accelerated the loss of redundant genes, especially newly duplicated ones.

## Discussion

Traditionally, the papaya cultivation population was classified based on fruit size, with common papaya having fruits larger than 1 kg and solo papaya having smaller fruits [15]. However, papaya breeding often requires hybridization of different varieties, and offspring of large-fruited accessions may also exhibit small fruits. Therefore, population classification based on fruit size is inconsistent with molecular-level differences. This study constructed a phylogenetic tree based on SNPs to reclassify 201 papaya accessions, providing valuable insights into the population structure at the molecular level. This population classification is based on genomic information and may differ from traditional methods, but it facilitates population genomics analysis of different groups. For instance, based on our classification, the solo population has the smallest π value, which may be due to a genetic bottleneck. This could be attributed to the fact that papaya plantations in the United States in the 20th century suffered from severe virus infections, significantly reducing yields [29]. On the other hand, the common population has undergone more hybridization with wild accessions, which can be observed in the results of the population structure analysis. Therefore, the diversity of the common population is higher than that of the wild, solo, and near-wild populations.

While cultivated papaya has a larger fruit size and richer flavor than wild papaya, it has a lower disease resistance. Yue *et al.* [15] analyzed selection signals between wild and cultivated papaya through population resequencing, identifying a significant selection of genes associated with flesh color (zeta-carotene desaturase) and some related to fruit sugar metabolism due to domestication. To get more insight, the present study integrated the WGS data of 49 papaya accessions. Growth- and stress-resistance-related genes in regions with domestication selection signals were identified. Among these genes, *Cp_zihui05794*, which encodes a WD40 domain protein, may play a significant role in regulating fruit growth or maturation. WD40 genes, such as *KRN2* in maize and its rice ortholog *OsKRN2*, have been found to be subject to convergent selection and negatively regulate crop yield by affecting tiller number [30]. In addition, WD40 proteins have been found to play an important regulatory role in tomato fruit ripening [31]. Due to the conservation of gene function across different species, *Cp_zihui05794* may play a regulatory role in fruit ripening and even the final yield traits of papaya.

While Yue *et al.*’s study investigated the population genomics of papaya, phenotype data collection and association analysis were not conducted [15]. This study identified multiple candidate genes related to yield by GWAS, including *Cp_zihui06549* (leucine-rich receptor-like protein kinase family protein) and *Cp_zihui06768* (accumulation of photosystem one, APO1). Furthermore, we heterogeneously expressed *Cp_zihui06549* in tomato and found that the number of fruits and branches in the overexpression line increased compared to wild-type tomato, which directly led to an increase in yield. Correspondingly, the ‘Zihui’ papaya with high expression of *Cp_zihui06549* exhibited more inflorescences and flowers. Papaya flowers are actinomorphic cymes arranged in inflorescences on the leaf-stem junction, and the number of inflorescences and flowers is an important factor affecting papaya yield. In previous study, we found that during the same cultivation period, the total number of inflorescences and flowers, as well as the fruit-setting rate of ‘Zihui’ papaya were higher than those of common papaya varieties, which directly led to a significant increase in fruit number and yield of ‘Zihui’ [16]. These results suggest that the high expression of *Cp_zihui06549* may promote the number of inflorescences and flowers, thereby increasing the yield of papaya. Moreover, previous studies have reported that members of the LRR-RKs family can act as a signaling receptor, potentially regulating crop yield by modulating responses to hormones [32, 33]. However, there are many factors that affect inflorescence and flower differentiation, and the mechanism is complex. The specific mechanism of *Cp_zihui06549* will be the focus of our future research. Therefore, *Cp_zihui06549* is a very important candidate gene that may provide guidance for the improvement of papaya. Another candidate gene, *APO1*, may play an important role in the formation of photosystem I [34]. And photosystems in fruit development can affect the net photosynthetic rate, influencing fruit size and yield [35], we will also conduct in-depth research on this gene in the future.

To further explore the genetic resources of papaya’s nonreference sequences, we constructed the pan-genome of papaya. There were 1530 genes annotated in the nonreference sequences, providing a more comprehensive gene resource. Genes with a lower frequency in cultivated species were identified, particularly genes related to ‘terpene synthase activity’, which play a vital role in plant resistance to pathogens and response to herbivorous feeding [36]. The reduction in gene frequency, especially the loss of genes in cultivated populations due to selective pressures and genetic drift, can impact the performance of crops [37]. The loss of genes associated with the synthesis of secondary metabolites from wild papaya in cultivated populations may be related to the resistance of papaya.

Analysis of population gene PAVs based on the pan-genome can provide new insights into papaya evolution and domestication. For example, accession-specific genes can be used to accurately identify specific accessions and infer hybridization processes of existing accessions. The number of genes in a redundant gene family has a dosage effect, and the number of functionally related genes can cause a gradient change in phenotype [38, 39]. Gene retention analysis can be performed using PAV analysis based on the pan-genome, as some genes undergo a period of gene retention following gene duplication. The Pearli4–1 gene identified in this study is an example that sheds light on our knowledge of gene replication and retention in papaya. It also provides a foundation for future research on papaya, such as the dosage effects of these genes, their biological functions, and the significance of functional differentiation of redundant genes.

This study conducted a comprehensive transcriptome analysis of papaya, demonstrating their expression patterns in multiple tissues. The highest proportion of shell genes expressed specifically in papaya fruits suggests that these genes may be subject to gene PAV selection. These genes under gene PAV selection may be associated with traits related to fruit development and ripening. Moreover, genes with leaf-specific expression are enriched in GO terms related to photosynthesis. These results indicate the importance of photosynthesis in papaya fruits. Cucumbers have mature green fruits, and the contribution of skin photosynthesis to their carbon accumulation is 9.4% [40]. Papaya fruits remain green for a long time, and the large skin area of the cultivated accessions may play a vital role in carbon accumulation. In summary, assembling the ‘Zihui’ genome, sequencing and analysis of different germplasm resources, construction of the pan-genome and PAV analysis, and analysis of RNA expression profiles provide a deeper understanding of papaya’s domestication process, genetic loci for agronomic traits, and some physiological characteristics. This study provides a ground work for more research and breeding of papaya.

## Materials and methods

### Plant materials

The main cultivated papaya ‘Zihui’, known for its high quality and high yield, was used in this study for de novo assembling. The plants were grown in Guangzhou, Guangdong Province, China, and leaf tissues collected and stored at −80°C. In addition to ‘Zihui’, 49 other papaya accessions were collected from China, and fresh leaf tissues were sampled and stored at −80°C. For RNA sequencing, tissues from the root, stem, leaf, flower, and fruit of ‘Zihui’ were collected and stored at −80°C.

### Genome and transcriptome sequencing

The papaya genome was sequenced using both second-generation sequencing (NGS) and third-generation PacBio sequencing platforms. For NGS, an HiC library was prepared from cross-linked chromatin in papaya cells according to the standard HiC protocol. RNA samples were extracted using the TRIzol kit from the roots, stems, leaves, flowers, and fruits of the same papaya plant used for DNA sequencing, mixed at the equimolar ratio, and then used to construct transcriptome sequencing libraries. In addition, genomic DNA samples were extracted using the cetyltrimethylammonium bromide (CTAB) method from leaf buds of the 49 papaya samples used for whole-genome resequencing, and then used to construct short-insert fragment libraries. These libraries were all sequenced on Illumina’s X Ten platform. For PacBio sequencing, papaya DNA samples were extracted using the AMPure bead cleanup kit (Beckman Coulter, High Wycombe, UK) following the manufacturer’s protocol. A SMRTbell library was constructed according to PacBio’s standard protocol and sequenced on a PacBio Sequel II instrument using Sequencing Primer V2 and Sequel II Binding Kit 2.0.

### Genome assembly and annotation

HiC data were used to assemble HiFi long-read fragments using Hifiasm [41] with—h1 and —h2 options to obtain a pair of haplotype-resolved assemblies and a pseudomolecule assembly. After obtaining the assembled contigs, we aligned second-generation sequencing (NGS) data from the same sample to the contigs using BWA [42] and polished the assembly for three rounds using pilon [43]. To further improve the scaffolding, we utilized RaGOO [44] to assemble contigs into pseudomolecules with two high-quality genomes, ‘SunUp’ and ‘Sunset’ [15], as references. To resolve conflicts resulting from the two versions of reference genomes, we aligned HiC data to the Hifiasm-assembled contigs using BWA and processed the alignment results using PreprocessSAMs.pl and filterBAM_forHiC.pl in ALLHiC [45, 46]. We then merged and reconciled the results obtained from the two reference genomes using HiC data alignment information with the merge function of ragtag (https://github.com/malonge/RagTag) [47].

We used RepeatMasker [48] to mask the repeat sequences and annotate the genome. In addition, we used RepeatModeler [49] to *de novo* identify repeat sequences and further annotated the genome using RepeatMasker. Tandem Repeats Finder was also used for repeat sequence annotation in the papaya genome [50].

An AUGUSTUS training model was generated using the annotated gene structure of the ‘Sunset’ papaya genome as input information [51] and used as the input information for the maker2 software [52]. RNA-seq data of ‘Zihui’ papaya were assembled using Trinity [53], and the assembled transcripts used as input files for maker2. Furthermore, protein sequences from *Actinidia_chinensis*, *Arabidopsis_thaliana*, *Camellia_sinensis*, and the ‘Sunset’ cultivar were also used as input files for maker2. The est2genome and protein2genome options were set at 1 in maker2 to predict gene structures using transcript and protein sequences. Annotation results for ‘Zihui’ obtained from maker2 were considered reliable if the annotation edit distance (AED) value was less than 0.5.

### Phylogenomic analysis and divergence time estimation

Whole genome protein sequences of 12 species, including *Arabidopsis thaliana*, *Arabis alpina*, *Brassica oleracea*, *Brassica rapa*, *Raphanus sativus*, *Thlaspi arvense*, *Citrus clementina*, *Malus domestica*, *Solanum lycopersicum*, *Vitis vinifera*, *Oryza sativa*, and *Zea mays*, were obtained from the NCBI Assembly database (https://www.ncbi.nlm.nih.gov/assembly/). OrthoFinder [54] was used to identify single-copy orthologs. Multiple sequence alignment of single-copy orthologs was conducted with MAFFT [55]. Next, ambiguous sites were trimmed with trimAl [56] using the ‘automated1’ option. A species tree was reconstructed with trimmed alignments using CASTER [57]. IQ-TREE [58] conducted a constraint search to estimate the branch lengths of the species tree. Finally, r8s [59] was used to infer absolute rates of molecular evolution and divergence times.

### Comparative genomics analysis

Blastp was used to compare the ‘Zihui’ genome to ‘Sunset’ and ‘SunUp’ genomes at the protein sequence level, with a threshold e-value of less than 1e-5. For genes unique to the ‘Zihui’ genome, hypergeometric analysis was used for GO enrichment analysis with a significance threshold of p.adj less than 0.05. JCVI was used to visualize chromosome collinearity [60].

### Resequencing data alignment and SNP calling

The present study performed whole genome sequencing of 49 accessions and retrieved resequencing data from 152 samples [14, 15, 17]. After quality control with fastp [61], the clean data were mapped to the ‘Zihui’ genome using BWA. HaplotypeCaller in GATK was used for SNP calling [62], followed by genotype calling using CombineGVCFs. To ensure the quality of SNP calling, SNP loci with a read coverage depth below 2× and above 50× were removed. Then, GATK’s variantFiltration with the parameters ‘--filter-expression “QD < 2.0 || FS > 60.0 || MQ < 40.0 || SOR > 3.0 || MQRankSum < -12.5 || ReadPosRankSum < -8.0”’ was used to further filter SNPs. Finally, vcftools [63] was used to remove SNP loci with a missing rate of more than 90% and an allele frequency of less than 5% in the papaya resequencing population.

### Selective signal analysis

To identify selective signals in different regions of the papaya chromosomes, we performed an XP-CLR test (version 1.0) [64] with ‘Zihui’ as the reference genome and compared 152 cultivated and 49 wild papaya accessions. The XP-CLR software was run separately for each chromosome with parameters set as -w1 0.0051002000 1 -p1 0.7. Each chromosome was divided into non-overlapping windows of 10 kb, and the average XP-CLR likelihood scores were calculated for each window. Regions with the top 5% average XP-CLR likelihood scores across all windows were considered to have strong selective signals. Adjacent or one intervening region between two other regions was merged into a new window, and the maximum average XP-CLR likelihood score was taken as the XP-CLR likelihood score for the new window. Then, π values of candidate genes in the selective signal regions were calculated in wild and cultivated papaya. Fst values were also calculated in the gene and its upstream and downstream 4-kb regions separately between wild and cultivated papaya.

### GWAS and gene haplotype analysis

We conducted phenotyping for 49 accessions. To perform SNP pruning, we first converted the VCF format file to the PED format using VCF tools and then to the BED format using PLINK [65]. We used the mixed linear model in EMMAX software for GWAS [66], with the Balding-Nichols kinship matrix and the first 10 principal component analysis (PCA) components as covariates. We analyzed the fruit weight and yield phenotype of 47 samples. We further identified significantly associated loci in gene or promoter regions and analyzed haplotypes associated with the phenotypes in these regions using CandiHap [67]. Analysis of the relationship between this haplotype and phenotype was conducted by selecting a panel of SNPs suitable for defining haplotypes surrounding the target gene and for evaluating variations in the influenced traits captured from the genomic region. Inter-group significance was assessed using the Wilcoxon test.

### Pan-genome construction and gene structure annotation

We extracted unmapped reads using SAMtools (v1.9) [68], with the parameters of ‘SAMtools fastq -f12’ for extracting paired-end reads that did not map to the reference genome, ‘SAMtools fastq -f 68 -F 8’ for extracting left-end reads that did not map to the reference genome, and ‘SAMtools fastq -f 132 -F 8’ for extracting right-end reads that did not map to the reference genome. We then used MaSuRCA [69] to assemble the unmapped reads from each sample and blasted the assembled contigs against the NCBI NT database using Blastn, and removed the unmapped contaminants, including sequences that were not in the superkingdom Eukaryota and those that were in the superkingdom Eukaryota but not in the kingdom Viridiplantae, based on the species information of the NT sequences. After merging all clean contigs obtained from the samples, we removed redundancy using cd-hit [70] and compared the nonreference sequences with the organelle genomes using nucmer to remove organelle genome contigs. Similar to the de novo assembled genome, we annotated the repeat sequence of the nonreference sequences using RepeatModeler and RepeatMask.

We obtained RNA-seq data from 129 samples of 8 bio-projects (PRJNA470602, PRJNA591254, PRJNA528193, PRJNA687615, PRJNA549650, PRJNA713527, PRJNA565901, and PRJNA880626) from NCBI, converted them to the fastq format using fastq-dump, and processed them using fastp to remove low-quality sequences. We then mapped the reads to the nonreference sequences using hisat2 [71], extracted the reads that mapped to the nonreference sequences using SAMtools, and assembled the transcripts using Trinity. Finally, we performed genome structure annotation of nonreference sequences with the assembled transcripts and protein sequences from multiple species as input files using the maker2 software.

### Gene PAV analysis

We used BWA to map the resequencing data to the papaya pangenome and determine the presence or absence of each gene using SGSGeneLoss v0.1 [72], with parameters ‘minCov = 2, lostCutoff = 0.2’, meaning that a gene was considered present if at least 20% of its exon regions had coverage of at least two reads and absent otherwise. Gene absence was denoted by ‘0’ and gene presence by ‘1’. Based on binary gene PAV data, we built a maximum-likelihood phylogenetic tree with 1000 bootstraps for the papaya populations using iqtree [58]. We also performed population structure analysis on the binary gene PAV data using STRUCTURE [73].

To investigate whether gene PAVs were under selection during domestication, we employed Fisher’s exact test to compare gene frequencies between cultivated and wild populations. We corrected multiple testing using the Benjamini–Hochberg method and considered genes with p.adj less than 0.001 and a fold difference in gene frequency greater than 2 to be under selection. We also conducted GO term enrichment of genes under selection using a hypergeometric distribution test.

### Transcriptome analysis

We aligned the RNA-seq data of the retrieved 129 samples to the ‘Zihui’ genome using hisat2. FeatureCounts [74] was used to calculate the read counts for each gene and subsequently converted the counts to FPKM values. We then performed PCA analysis on samples from different organs or tissues using the R function rda in vegan package [75]. We further identified differentially expressed genes (DEGs) using the R package DESeq2 with a threshold of p.adj < 0.05 and fold change >2 [76]. To further evaluate the tissue- or organ-specific expression of genes, we computed the tissue or organ specificity index using the following formula:


$$ tau=\frac{n}{n-1}-\frac{\sum_{i=1}^n{x}_i}{\left(n-1\right)\times \underset{1\le i\le n}{\max}\left({x}_i\right)} $$

where n represents the number of tissues or organs, *x* represents the mean expression of a gene across samples belonging to the same tissue or organ, and *i* represents a particular tissue or organ. The specificity index is ranged from 0 (broad expression) to 1 (restricted expression). For genes with tissue-specific expression, we further performed hypergeometric analyses to identify enriched GO terms.

### Gene retention analysis

Using pfam annotation of protein sequences, we identified shell and core genes containing the same pfam and identified putative paralogs with similar sequences, some of which may have undergone gene loss in many individuals during evolution. To determine the transcriptional response patterns of these genes, we analyzed the selected genes in the above transcriptome data. We also conducted pfam annotation and blast alignment of these genes in 11 other species. Finally, we performed a multiple sequence alignment of the protein sequences from multiple species using mafft [77] and constructed a maximum likelihood phylogenetic tree using iqtree.

### Heterologous expression of *Cp_zihui06549* in tomato

The CDS of *Cp_zihui06549* was recombined with the p2301–35SN-GFP vector using primers *Cp_zihui06549*-LP/*Cp_zihui06549*-RP (Supplementary Table S9). The recombinant plasmid (p2301–35SN-GFP-Cp_zihui06549) was transformed into *Agrobacterium tumefaciens* (EHA105) for wild-type tomato (‘Micro-Tom’) genetic transformation. Tomato (‘Micro-Tom’) leaves were selected, their tips and petioles removed, and 0.5 × 0.5 cm pieces removed as explants for genetic transformation. Heterologous expression in the tomato experiment was conducted as described by Tang *et al.* [78]. The positive transgenic plants were identified by PCR detection. The T1/T2 seeds from each transgenic line were screened with MS medium that containing 25 μg ml^−1^ hygromycin. A total of 10 independent transformed homozygous transgenic lines (LRR-RLK OE1–10) were obtained. Young leaves from the 10 independent lines were collected and used as materials to detect the transcription level of *Cp_zihui06549* in each independent line by RT-PCR. The specific primers *Cp_zihui06549*-1LP/*Cp_zihui06549*-1RP are listed in Supplementary Table S9. Three transgenic lines (OE1, OE2, and OE3) were selected for further phenotypic analysis. Ten plants were used for phenotype and statistical analysis for each transgenic line. The experiment was conducted in three biological replicates. The statistical calculation methods for single fruit weight and single plant yield were as follows: for single plant yield, the weight of the first to last mature fruit was continuously measured and statistically analyzed throughout the entire ripening period of each plant. For the weight of a single fruit, the weight of the first 20 fully developed and mature fruits within the same growth period after flowering on each plant was measured and statistically analyzed. The experiment was conducted with three biological replicates and 10 plants per independent line per biological replicate.

### qRT-PCR analysis

Total RNA was extracted from various organs using the RNeasy plant mini kit (Qiagen) and reverse transcribed into cDNA by the HiScript II Q Select RT SuperMix (Vazyme Biotech). qRT-PCR was carried out according to our previous description [3]. The relative gene expression level was calculated by the 2^-ΔΔCt^ method. Eukaryotic initiation factors 4A (*EIF4A*) were used as internal controls. The Primer Premier 5.0 was used to design primers for qRT-PCR. The primers are listed in Supplementary Table S9.

### PCR verification

To verify the gene PAV calling results, PCR assays were performed on the same DNA samples originally used for genome resequencing. PCR was performed on the T100 Thermal Cycler System using KOD FX polymerase (TOYOBO) according to the manufacturer’s instructions. Twelve samples and five genes were randomly selected for PCR detection. The genes’ specific primers were designed using Primer Premier 5.0 software (Supplementary Table S9).

#### Statistical analysis

All experiments in our study were repeated at least three times. All data were analyzed using SPSS software version 21.0. We used the Student’s *t*-test to analyze the difference between control and treatment groups. We designated a *P*-value of less than 0.05 as a significant difference.

## Supplementary Material

Web_Material_uhaf045

## Data Availability

The Hi-C, HiFi, RNA-Seq and genome assembly data were deposited in the National Centre for Biotechnology Information (NCBI) under BioProject ID PRJNA968045. The whole-genome resequencing was deposited in the National Centre for Biotechnology Information (NCBI) under BioProject ID PRJNA970517. The nonreference contigs of *Carica papaya* pangenome and annotation are available at Figshare database. (https://figshare.com/articles/dataset/Carica_papaya_pangenome/22887086).
