# Supplementary figures and images for: Genomic insights into the domestication and genetic basis of yield in papaya
Source: Hortic Res. 2025 Feb 17;12(5):uhaf045. doi: 10.1093/hr/uhaf045 (PMC11997427; doi:10.1093/hr/uhaf045)

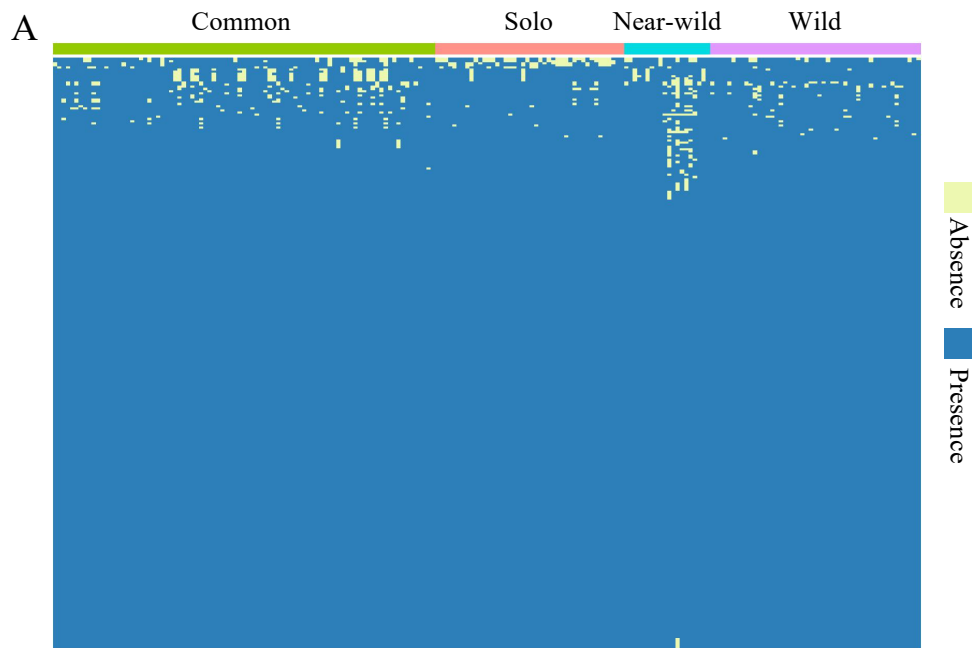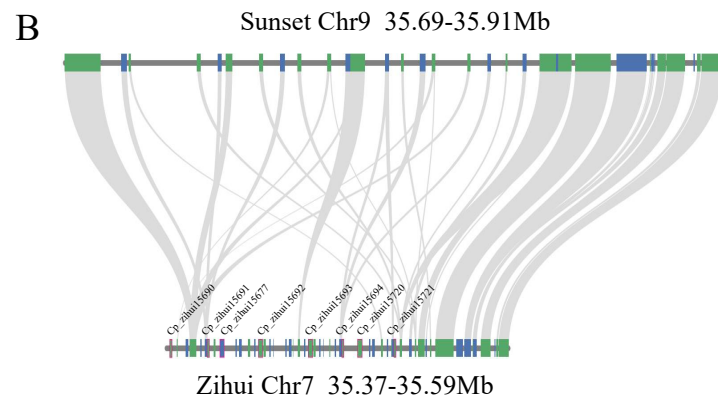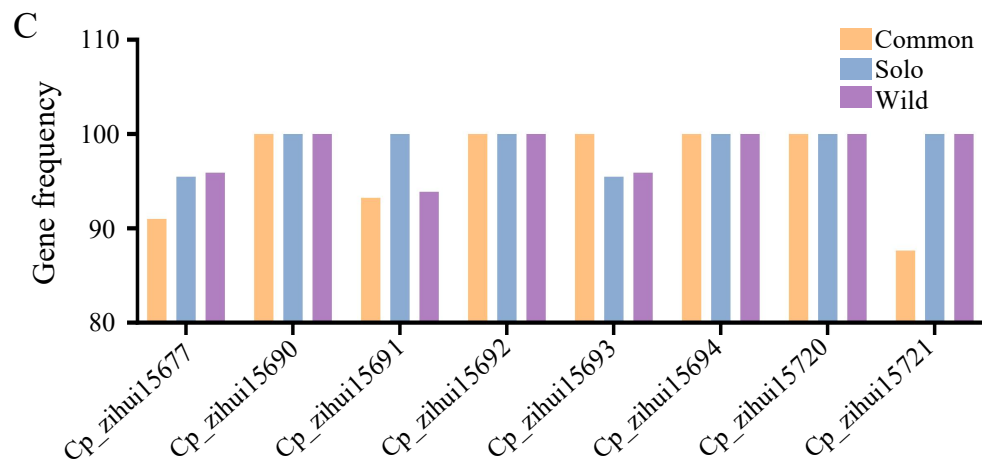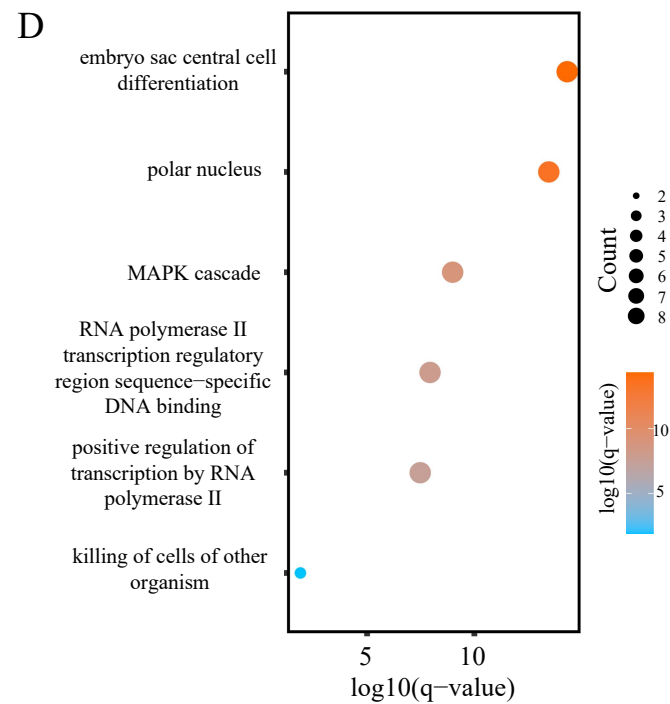

Supplement: Web_Material_uhaf045 [file web_material_uhaf045.zip › Supplementary Figure 1.pdf]

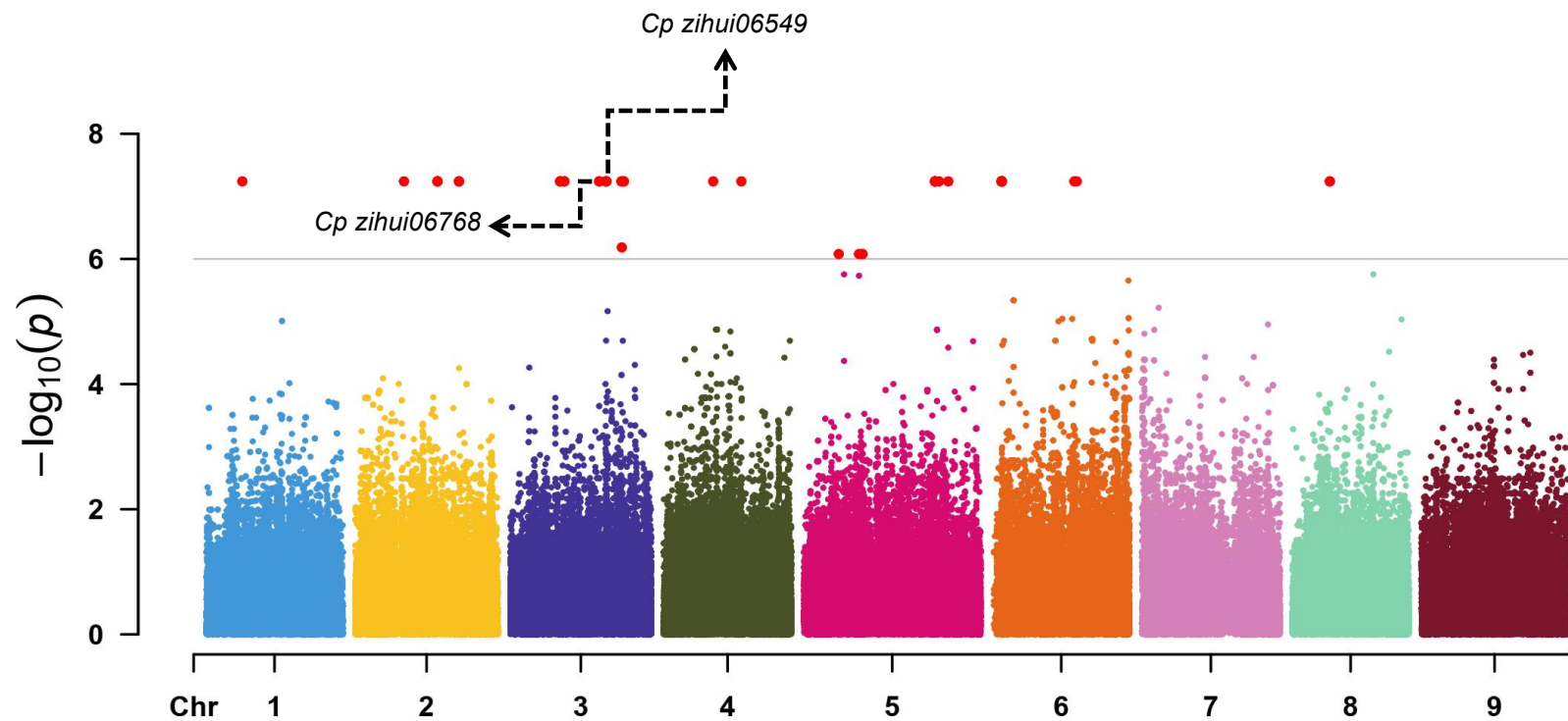

Supplement: Web_Material_uhaf045 [file web_material_uhaf045.zip › Supplementary Figure 2.pdf]

A

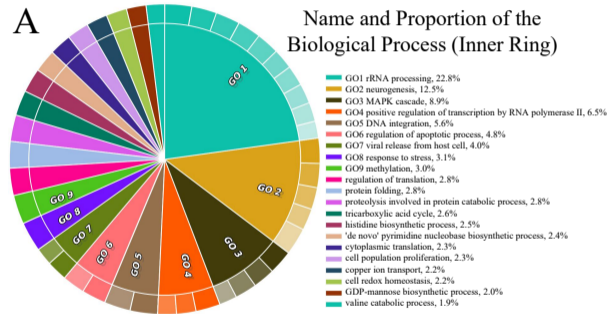

B

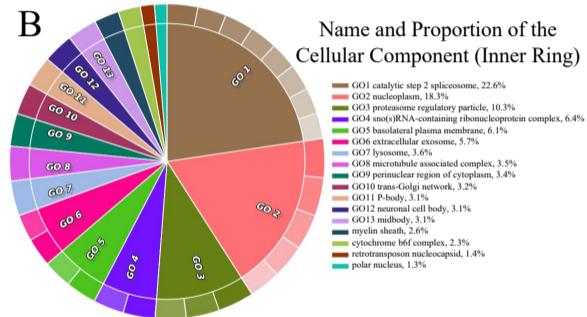

Supplement: Web_Material_uhaf045 [file web_material_uhaf045.zip › Supplementary Figure 3.pdf]

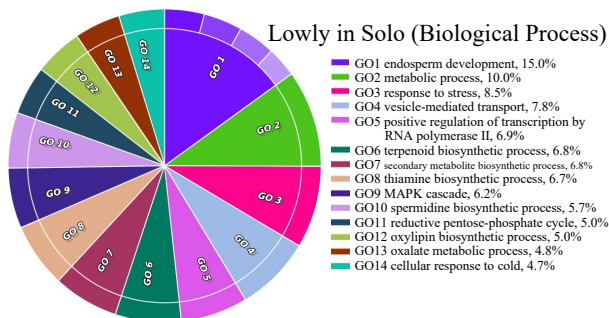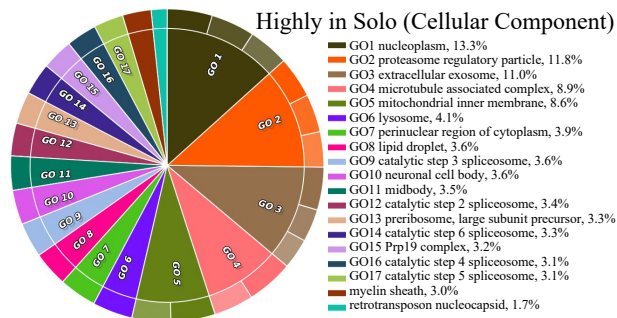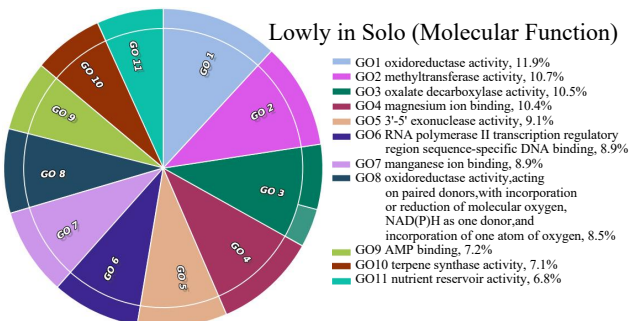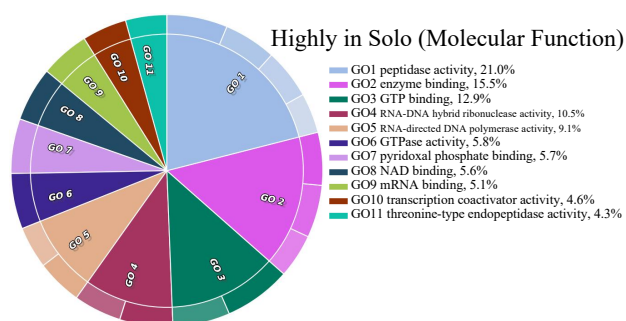

Supplement: Web_Material_uhaf045 [file web_material_uhaf045.zip › Supplementary Figure 4.pdf]
